# The Discoverer of the Effects of Sulphuric Ether

**Published:** 1847-01

**Authors:** P. W. Ellsworth


*The Discoverer of the Effects of Sulphuric Ether.* By P. W. Ellsworth.—We find, by an advertisement in one of the papers that Drs. C. T. Jackson and W. T. G. Morton have made an important invention which has been patented. In justice to a fellow townsman, I will give its true history. The first announcement publicly made was by myself, more than a year since, in an article written for the purpose of establishing the doctrine that in disease the vital power is diminished, and suggested that in all probability pain was but a peculiar depressed state of the sensor nerves, and in proof stated that stimulants acting upon this system, and having a certain relation to it, would relieve or prevent suffering; and that the dentists of Hartford were in the habit of administering nitrous oxide gas, which enabled them to extract teeth without the consciousness of the patient. The original discoverer of this was Horace Wells, dentist in this city, and he tried the first experiment upon himself. After the idea suggested itself to him, he debated for some time which to use, the gas or ether, but preferred the former as he thought it less liable to injure the system. Being now satisfied of its powers, he went to Boston for the sole purpose of introducing it to the faculty. He presented it to Dr. Warren, who laid it before his class, but the experiment first attempted partially failing, and no one seeming willing to lend him an helping hand, he ceased making any further personal efforts. He especially made known his discovery to Drs. Jackson and Morton, neither of whom had any idea of it until this moment, and must allow Dr. Wells the whole merit of the thing up to this point. We see by the Journal that Drs. J. and M. call their invention a peculiar compound. I was fully satisfied that sulphuric ether was *the* article, as it was known to be among the ingredients, and being there, nothing else was wanting to produce the desired effect. The claim, as published, sets the matter at rest; ether, and ether alone, is used, and the world will easily judge how much right Drs. Jackson and Morton have to patent it. Had they been the first to discover the fact that any gas would produce exemption from pain, and had made it known, they would have deserved commendation. They have not done this, nor justice to the true discoverer. Is there any merit in using ether in place of nitrous oxide gas? Certainly not, for the properties of the two things are so alike in this respect, that one is constantly used for the other, and for months I supposed our dentists were using both; and the idea of allowing any man a patent for the use of the one after the effects of the other were known, is preposterous. Dr. Wells's experiments were numerous and satisfactory. One fact discovered, is extremely interesting. It is that however wild and ungovernable a person may be when taking the gas, simply for experiment, he becomes perfectly tranquil when it is inhaled before an operation: that the mind being prepared, seems to keep control over the body, indisposing to any effort.

Unfortunately it is too true, that mystery, as of a nostrum, is frequently required to induce people, and sometimes the profession, to notice an improvement, and thus far perhaps thanks are due Dr. Morton for compelling attention; yet we must give Dr. Wells the credit he justly deserves of making the discovery, spending time and money in its investigation, and then in nobly presenting it to the world. It is to be hoped every other gas and substance capable of exciting the nervous system may be experimented upon, but we hope no one will think of patenting any if discovered to be similar in its operation.—*Boston Med. and Sur. Journ.*

